# Cystic degeneration of neuro endocrine tumor of pancreas and Crohn’s disease: true or coincidental association?

**DOI:** 10.11604/pamj.2016.25.7.9524

**Published:** 2016-09-15

**Authors:** Mohamed Hedfi, Imed Abbasi, Chibani Intissar, Ammar Salwa, Adnen Chouchen

**Affiliations:** 1 Department Of Surgery, FSI Hospital, Marsa, Tunisia

**Keywords:** cystic neoplasms, pancreatic endocrine tumours, Crohn´s disease, inflammatory bowel disease

## Abstract

Pancreatic endocrine tumors (PETs) or islet cell tumors are rare lesions, the incidence of which is estimated to be less than 1 per 100,000 person-years in the general population. PETs can be divided into functional (exhibit a distinct clinical syndrome due to hormone hypersecretion) and non-functional tumors. The majority of PETs are non-functional. In spite of their rarity, cystic neoplasms of the pancreas are characterized by existing or potential malignancy that cannot be ignored during decisive process with regard to the choice of treatment. The purpose of this workis to find an association with Crohn's disease and cystic degeneration of a neuroendocrine tumor of the pancreas. Crohn's disease may affect extraintestinal organs, including the pancreas. In such cases, It seems certain that many patients diagnosed with Crohn disease (CD) are predisposed to a wider spectrum of cancers. We present a case of pancreatic cyst with no typical features of pseudocyst in the medical interview, with history of Crohn's disease, treated by caudal pancreatectomy. We tried to evaluate the clinical and morphological features of so-called cystic neoplasms associated with inflammatory bowel disease and to define their pathological characteristics.

## Introduction

An increased risk of cancer in Crohn's disease was observed in population studies. This risk is primarily characterized by an increase in the incidence of adenocarcinoma of the colon and small bowel. The significant association of other types of cancer has never been shown in Crohn's disease. A few years ago, University of Utah researchers say they noticed that pancreatic cancer seemed to be developing at higher-than-normal rates in IBD patients and their family members. CD is an autoimmune disease and we hypothesize that the patients are predisposed to a wider spectrum of cancers [1]. To see if there was an association, were port the case of a patient with a neuro-endocrine tumor of pancreas degenerated and appeared during the evolution of a Crohn's disease.

## Patient and observation

**Figure 1:**
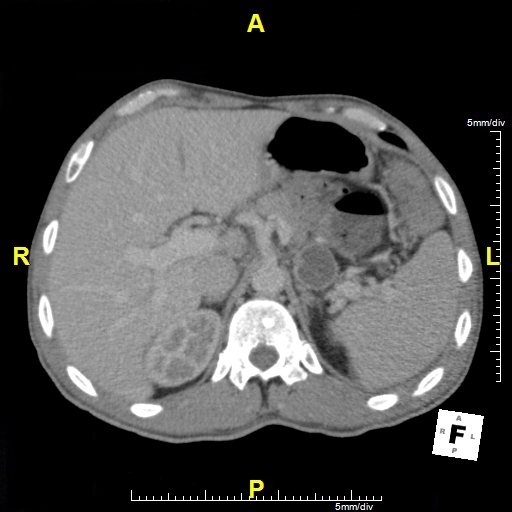
CT scan showing cystic lesion of the pancreas. Thin walled lesion without segmentation with homogenous fluid content

**Figure 2:**
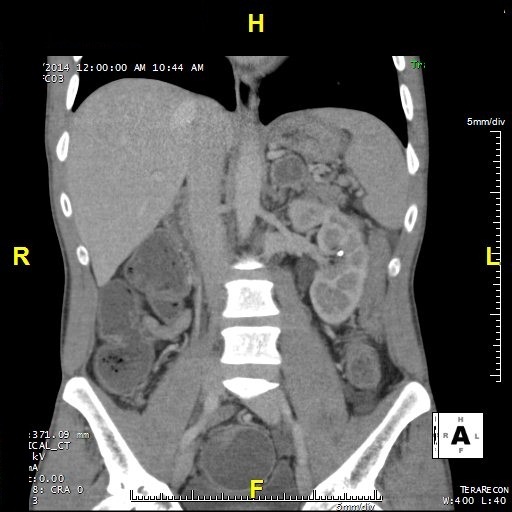
CT scan longitudinal view pancreatic cyst located in the tail of pancreas

We present a case of pancreatic tumor without a history of trauma or panceratitis. A 47-year-old Tunisian man with a history of Crohn's disease was admitted to the University Hospital in 2015 because of fluid chronic diarrhea with 4 stools per day daytime only with out ooddebr is associated with vomiting with out abdominal pain or fever with a weight loss not encrypted dating from 6 months. Laboratory tests were normal. Nonspecific elevations of serum pancreatic enzymes. Patient underwent an abdominal ultrasound and computed tomography (CT) that revealed: Aspect of ileitis of the last ileal loop extended by 300 mm with distended ileal loops upstream, cystic image at the tail of the pancreas with clean wall uncalcified and hypodense content of fluid density without endoluminal bourgeon or pancreatic duct dilatation, measuring 28 x 22mm (Figure 1). The size of the head was normal. There were no enlarged lymph nodes (Figure 2). Aspect of bilateral sacroiliitis and bilateral coxarthrosis. MRI show an aspect of ileitis of the last ileal loop and the cystic nature of a pancreatic lesion, with parietal enhancement at the caudal portion of the pancreas with a slight dilation of the Wirsung. Otherwise, gallbladder contains multiple gallstones and biliary sludge. Tumor markers are normal.on the other hand, in colonoscopy terminal ileum appears swollen, inflamed, with frequent ulcerations and biopsies were made. The biopsies were taken and show a subacute and ulcerative ileitis compatible with Crohn's disease. Esophagogastroduodenoscopy was normal. The patient was operated by laparoscopy, he had a caudal pancreatectomy and cholecystectomy .1.5 cm cystic tumor in the tail of pancreas was resected and sent for pathological examination. The post-operative course was uneventful. Patient was discharged home within 7 days in a good general condition. There were no symptoms of glucose intolerance after normal diet administration. The microscopic examination revealed well differentiated cystic neuro endocrine tumor of the tail of the pancreas grade I according to the classification of WHO 2010 (mitosis <2/10CFG and Ki67<2% ) associated with neuroendocrine hyperplasia lesions of islets of Langerhans, surgical limit was healthy, pTNM pT2, ectopic spleen, chronic cholecystitis. the patient come for out-patient consultations at the hospital, he felt well, with no severe complaints and with correct periodic findings and normal glucose metabolism.

## Discussion

Neuroendocrine tumors account for only 1-2% of all pancreatic neoplasms and less than 10% of all neuroendocrine tumors are cystic, which makes cystic neuroendocrine lesions very rare [2]. The mean age at diagnosis is 53 years and there is no evident gender predilection [3, 4]. Most cystic neuroendocrine tumors in the pancreas are non-functional and the diagnosis is usually made incidentally or secondary to mass-dependent symptoms, such as abdominal pain, nausea or weight-loss, as was the case in our patient [3]. Pancreatic neuroendocrine tumors rarely undergo cystic degeneration leading to a radiologic appearance, which is usually confused with other cystic pancreatic tumors, such as mucinous cystadenoma, mucinous cystadenocarcinoma, serous cystadenoma, and solid and cystic papillary tumors, or with non-neoplastic pseudocysts [5]. The clinical manifestation of cysts and cystic tumors can be identical or they often can be asymptomatic. Symptoms as abdominal discomfort or abdominal pain, nausea, vomiting, diarrhea, fever and leukocytosis, icterus, recurrence pancreatitis, bleeding from the digestive tract, abdominal mass are typical in both pseudocyst and neoplasm cases [6]. In our case, there are no specific symptom orientate to pancreatic tumor, no symptoms in the physical examination were manifested. Furthermore, the patient was without a history of trauma or panceratitis. Cystic pancreatic lesions are more and more frequently found due to the large use of imaging procedures during systematic checkup, workup of abdominal pain, or follow-up of a patient with a non-pancreatic malignancy. The frequency of cystic pancreatic lesions was 20% in 1,444 American people who had a systematic magnetic resonance imaging (MRI) [6–8]. CT is found to be a very helpful tool in detection of pancreatic tumors, with sensitivity over 90%, but is not reliable for distinguishing neoplasm from pseudocyst, serous from mucinous tumors or benign from malignant. Also, tumors markers might be helpful in decision making and postoperative management of pancreatic neoplasms. Our patient had a normal preoperative tumors markers so didn't need to control them [8]. Except for neuroendocrine microadenomas, all neuroendocrine tumors are considered to have malignant potential and should be considered for surgical resection [5, 7]. During the last years, a considerable increase in interest concerning the relationship between IBD and pancreatic involvement has been noticed. A number of papers have been published referring to either concurrent appearance of acute pancreatitis and CD or UC patients, and/or to case series description, but no studies have established a causal relationship between Crohn's disease and pancreatic tumor [5, 8]. Many patients with autoimmune diseases, including those with CD, ulcerative colitis, rheumatoid arthritis, systemic lupus erythematosus and sarcoidosis, are at a risk of cancer. We hypothesized that the risks should be shown at many sites, typical of an autoimmune disease. The spectrum of cancers that were increased after CD included many sites that have not been reported before: liver , pancreas, lung, prostate, testicular, kidney and (squamous cell) skin cancers; endocrine tumors and leukemia[1, 5, 8].

## Conclusion

Crohn's disease is no longer considered an isolated gastrointestinal disorder, but rather a multiorgan disease. This case report confirms that Crohn's disease can affect the pancreas and may be responsible for its initial presentation. Crohn's disease should be included in the differential diagnosis of patients with unexplained pancreatitis. Biochemical, imaging, and functional studies of the pancreas may be indicated in patients with Crohn's disease and unexplained symptoms. According to our findings, we can suggest that there may be a link between Crohn's disease and pancreatic cancer and they could open the door for new screening recommendations for pancreatic cancer in patients with IBD and their families.
